# A clinical evaluation of VO_2_ kinetics in kidney transplant recipients

**DOI:** 10.1007/s00421-021-04672-x

**Published:** 2021-04-03

**Authors:** Alessandro Patti, Daniel Neunhaeuserer, Sara Ortolan, Fausto Roman, Andrea Gasperetti, Francesca Battista, Caterina Di Bella, Stefano Gobbo, Marco Bergamin, Lucrezia Furian, Andrea Ermolao

**Affiliations:** 1 Sport and Exercise Medicine Division, Department of Medicine, University Hospital of Padova, Via Giustiniani 2, 35128 Padua, Italy; 2 Kidney and Pancreas Transplant Unit, Department of Surgical, Oncological and Gastroenterological Sciences, University Hospital of Padova, Via Giustiniani 2, 35128 Padua, Italy

**Keywords:** Kidney transplantation, Oxygen uptake kinetics, Cardiopulmonary exercise testing, Haemoglobin, Muscle

## Abstract

**Purpose:**

Aerobic exercise capacity is reduced in patients with chronic kidney disease, partly due to alterations at the muscular and microvascular level. This study evaluated oxygen uptake (VO_2_) kinetics as indicator of muscular oxidative metabolism in a population of Kidney Transplant Recipients (KTRs).

**Methods:**

Two groups of KTRs enrolled 3 (*n* = 21) and 12 months (*n* = 14) after transplantation and a control group of healthy young adults (*n* = 16) underwent cardiopulmonary exercise testing on cycle-ergometer. The protocol consisted in two subsequent constant, moderate-load exercise phases with a final incremental test until exhaustion.

**Results:**

The time constant of VO_2_ kinetics was slower in KTRs at 3 and 12 months after transplantation compared to controls (50.4 ± 13.1 s and 43.8 ± 11.6 s vs 28.9 ± 8.4 s, respectively; *P* < 0.01). Peak VO_2_ was lower in KTRs evaluated 3 months after transplantation compared to patients evaluated after 1 year (21.3 ± 4.3 and 26.4 ± 8.0 mL/kg/min; *P* = 0.04). Blood haemoglobin (Hb) concentration was higher in KTRs evaluated at 12 months (12.8 ± 1.7 vs 14.6 ± 1.7 g/dL; *P* < 0.01). Among KTRs, τ showed a moderate negative correlation with Peak VO_2_ (ρ = − 0.52) and Oxygen uptake efficiency slope (OUES) (*r* = − 0.57) while no significant correlation with Hb and peak heart rate.

**Conclusions:**

KTRs show slower VO_2_ kinetics compared to healthy controls. Hb and peak VO_2_ seem to improve during the first year after transplantation. VO_2_ kinetics were significantly associated with indices of cardiorespiratory fitness, but less with central determinants of aerobic capacity, thus suggesting a potential usefulness of adding this index of muscular oxidative metabolism to functional evaluation in KTRs.

**Supplementary Information:**

The online version contains supplementary material available at 10.1007/s00421-021-04672-x.

## Introduction

Scientific evidence clearly shows that cardiorespiratory fitness is a strong predictor of all-cause and cardiovascular mortality independent of age, sex, ethnicity, and comorbidities (Myers et al. 2002; Kodama et al. 2009; Mandsager et al. 2018). Patients with chronic kidney disease (CKD) have generally reduced maximal aerobic exercise capacity compared to healthy subjects, which appears to slowly improve in Kidney Transplant Recipients (KTRs), even though often not reaching normal values (Kempeneers et al. 1990; Painter et al. 2002, 2011; Habedank et al. 2009). There are multiple reasons for this impairment in patients with CKD, since this population may present both central and peripheral limitations to exercise. As a matter of fact, anaemia, autonomic, vascular and cardiac dysfunction are common in patients with end-stage renal disease, frequently associated with skeletal muscle and/or metabolic abnormalities (Painter et al. 2011). In particular, muscular alterations have been reported in this population such as reduced capillary density, increased diffusion distance and reduced mitochondrial density and/or function (Kempeneers et al. 1990; Moore et al. 1993; Painter et al. 2011). These characteristics may explain the difficulty in restoring these patients’ aerobic capacity to normal values after transplantation, despite an increase in haematocrit and cardiac output, obtained with erythroid-stimulating agents and exercise training (Stray-Gundersen et al. 2016), should have a positive impact on maximal oxygen uptake (Johansen et al. 2010). Moreover, also kidney transplantation itself has been shown to facilitate the recovery of some central limitations to exercise. Indeed, with the normalization of renal function after transplantation the reduced blood haemoglobin (Hb) concentration and maximal heart rate may improve (Painter et al. 2011). Furthermore, deteriorations at the muscular level in KTRs might in part also be due to immunosuppressive therapy (Painter et al. 2003; Topp et al. 2003). Nevertheless, an improvement in muscle quality has been hypothesized (Habedank et al. 2009), and muscle alterations have been shown to improve after transplant with withdrawal from immunosuppressive therapy (Topp et al. 2003). The extent of muscle recovery has, however, not yet been elucidated and patients often maintain a condition of frailty and increased risk of falling (McAdams-DeMarco et al. 2017; Zanotto et al. 2017, 2020).

The kinetics of oxygen uptake (VO_2_ kinetics), i.e., the rate of adjustment of the oxygen consumption to a sudden increase in workload during the transition from rest to constant, moderate intensity exercise, is thought to reflect the oxygen utilization at a peripheral muscular level (Grassi 2005; Poole and Jones 2012). Reboredo et al. previously evaluated VO_2_ kinetics during a moderate intensity constant load in patients with CKD before and after a symptom-targeted intra-dialytic training program. The results of their study showed an improvement of this parameter with exercise training (Reboredo et al. 2015). To our knowledge, very limited data exist, specifically assessing VO_2_ kinetics in KTRs, particularly for different follow-up periods. The aim of the present study was thus to investigate VO_2_ kinetics in this population, to evaluate possible peripheral limitations to exercise that may have an impact on the typically lower exercise tolerance and cardiorespiratory fitness of these patients. Since important cardiopulmonary adaptations take place after renal transplant, we decided to evaluate this parameter 3 and 12 months after the surgical intervention. The secondary aim of the study was to evaluate if the VO_2_ kinetics were conditioned by other exercise-related variables in this specific population of KTRs.

## Methods

This study included KTRs who received transplant at the University Hospital of Padova between 2017 and 2018. Ethical approval of the experimental design was obtained from the Ethics Committee of the University of Padova (approval number 43079). All procedures were conducted in accordance with the Declaration of Helsinki and patients provided written informed consent. The evaluation was performed during routine clinical exercise testing, as previously described for other patient population (Neunhaeuserer et al. 2017, 2020).

A first group of KTRs included 21 patients evaluated 3 months after transplant, while the second group included 14 patients evaluated 1 year after transplant. Patients with significant systolic heart failure were excluded from the study. 16 young and apparently healthy subjects were enrolled as control group for analysis of VO_2_ kinetics. The baseline characteristics of the three groups are described in Table 1. The underlying CKDs of the included KTRs were of different aetiologies. Table 2 reports the main clinical features of the two groups of KTRs and shows that there was no difference in blood creatinine concentration, while Hb was significantly higher in the group evaluated 12 months after surgery.

**Table 1:** Characteristics of the study participants

	3-month group	12-month group	Control group
Number (*n*)	21	14	16
Male/Female (*n*)	13/8	12/2	9/7
Age (years)	53.52 ± 10.24^**^	50.29 ± 5.77^**^	26.18 ± 3.41
Height (cm)	168.96 ± 8.01	171.25 ± 9.46	173.88 ± 8.21
Weight (kg)	67.97 ± 11.51	74.56 ± 11.83	68.15 ± 10.48
BMI (kg/m^2^)^a^	23.72 ± 2.79	25.35 ± 2.76^**^	22.44 ± 2.14

The characteristics of the three groups are expressed as mean ± SD unless otherwise noted

BMI: Body mass Index

**Significantly different (*P* < .05) from value obtained in control group

^a^Data are non-normally distributed in at least one of the three groups

**Table 2:** Characteristics of the two groups of kidney transplant recipients

	3-month group	12-month group	*P*
Number	21	14	
Male	13 (62%)	12 (86%)	
Creatinine (μmol/L)	115.95 ± 22.92	123.36 ± 23.41	.360
Hemoglobin (g/dL)	12.77 ± 1.67	14.55 ± 1.75	.005
Β-blocker therapy	12 (57%)	6 (43%)	.407
Type and duration of dialytic therapy			
Dialytic therapy	18 (86%)	12 (86%)	1.000
Hemodialysis	8 (38%)	8 (57%)	.268
Peritoneal dialysis	7 (33%)	3 (21.5%)	.704
Hemodialysis + peritoneal dialysis	3 (14,5%)	1 (7%)	.653
Mean Dialysis Time (months)*	30.9 ± 33.6	24.9 ± 21.0	.904
Cause of chronic kidney disease			
Chronic glomerulonephritis	3 (14%)	–	
Polycystic kidney disease (APKD)	4 (19%)	5 (36%)	
Diabetic nephropathy	3 (14%)	–	
Lupus nephropathy	1 (5%)	–	
Malformation/renal genetic syndrome	–	2 (14%)	
Other or unknown	8 (38%)	5 (36%)	
Vasculitis	–	1 (7%)	
Hypertension	2 (10%)	1 (7%)	

Parameters are expressed as mean ± SD or n (%)

*One patient has been excluded from the present analysis because the length of his dialytic treatment was not known

### Exercise testing protocol

**Fig. 1:**
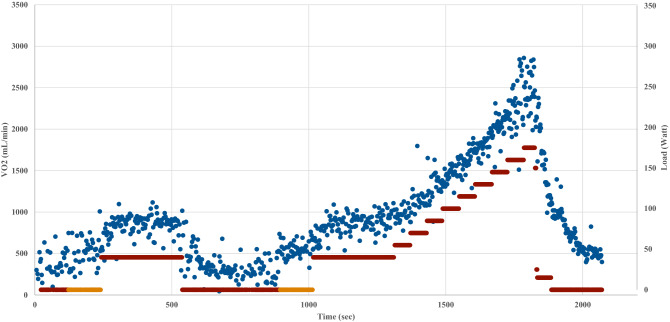
The exercise test protocol used for this study and the related oxygen consumption (VO_2_) of one of the participants. The dots represent breath-by-breath data of the subject’s VO_2_ during the test. The dark (red) bars display the exercise intensities in Watts during the different test phases, while the light (orange) bar shows the unloaded pedaling, performed before the constant load exercise test

For each patient medical history was taken, physical examination was performed and recent blood Hb and creatinine concentration were obtained. Cardiopulmonary exercise testing was subsequently performed on a cycle ergometer (eBike, General Electrics). To overcome the possible drop-outs that could have arisen testing patients on two different days, a specific protocol was designed to obtain both constant load and incremental exercise testing data in a single clinical evaluation (Fig. 1). The protocol consistent of two 5-min constant load tests, both preceded by 2 min of unloaded pedalling and separated by 6 min of resting. At the end of the second constant load exercise, an incremental test until exhaustion was carried out. Given the differences in the age and fitness level between KTRs and healthy subjects, the test protocols were slightly different: a constant load of 30 or 40 W was used for patients 3 months after transplant, a constant load of 40 W was used for patients 12 months after surgery and a constant load of 60 (female) or 75 (males) Watts was used for controls. These loads were chosen to be reasonably below the first ventilatory threshold (VT1) of the subject being tested, to avoid the occurrence of a slow component of VO_2_ kinetics (Poole and Jones 2012). The final incremental test consisted in a 15 W per minute ramp for patients, and a 25 W per minute ramp for controls. Study participants were asked to keep a constant pedal cadence of 60 ± 5 rpm (70 ± 5 rpm for the control group). Breath by breath cardiopulmonary parameters and 12 lead ECG were recorded during the whole test (Jaeger-Masterscreen-CPX, Carefusion, Germany).

### Data analysis

The first two constant load bouts were used for the determination of the VO_2_ kinetics during the transition from unloaded pedalling to moderate intensity exercise. First, the VO_2_ responses were linearly interpolated using a mathematical software (MATLAB R2017a, Math Works, MA, USA) to provide one value of VO_2_ per second; thereafter the values of the two bouts were averaged. Iterative nonlinear regression (Levenberg–Marquardt procedure) was used to characterize the primary VO_2_ response after removing the first 20 s of data (to eliminate the phase 1 component, known as the “cardiodynamic phase”), fitting the resting 5 min in a mono-exponential function according to the following equation:

$$\text{VO}_{2} = \text{ VO}_{2} ( \text{bl} ) + \text{A}^*(1-e^{-(t-\text{TD})/\tau})$$

where VO_2_(t) is the VO_2_ at time *t*; VO_2_(bl) is the baseline VO_2_ measured in the 30 s preceding the transition to exercise; and A_p_, TD_p_, and τ are the amplitude, time delay, and the time constant of the primary (phase 2) response of VO_2_ kinetics, respectively.

VO_2_ at peak exercise (peak VO_2_) was determined during a final, incremental ramp testing until patients’ exhaustion, defining peak VO_2_ as the highest average VO_2_ recorded during a 30-s period of testing. The first ventilatory threshold (VT1) was estimated using the V-slope method and evaluating the ventilatory equivalent of VO_2_ (Schneider et al. 1993). The oxygen uptake efficiency slope (OUES) was calculated as the coefficient of the linear relationship between oxygen uptake and the logarithm of total ventilation.

### Statistical analysis

Normality of variables was checked using the Shapiro–Wilk test. For comparisons between two groups, a chi-square test was used to assess the differences between categorical variables. In the case expected cell frequencies were greater than five a Fisher’s exact test was used. For continuous variables, a *t*-test was used for normally distributed variables and a Mann–Whitney U test for non-normally distributed variables. For comparisons between the three groups, one-way analysis of variance (ANOVA) with post hoc analysis (Tuckey’s post hoc test) was performed on all normally distributed variables. For parameters that violated the assumption of homogeneity of variances, a Welch test was performed and Games-Howell post hoc tests were used for multiple comparisons. For variables that showed a non-normal distribution in at least one of the three groups, a Kruskal–Wallis H test was performed and pairwise comparisons were conducted using Dunn’s (1964) procedure with a Bonferroni correction for multiple comparisons. A statistical significance level of 0.05 was used for all tests; pairwise comparisons results were expressed as adjusted P values. Controlling data for outliers, the analysis was performed also removing the outliers and their presence was tolerated if they did not alter the significance of the results obtained. To assess correlations between variables, Pearson’s r or Spearman’s rho correlation indexes were used for normally and non-normally distributed variables, respectively. Statistical analysis was performed using SPSS (v 25; IBM Corporation, Armonk, NY).

## Results

The parameters of cardiorespiratory fitness obtained during the incremental phase of cardiopulmonary exercise testing were significantly reduced in both groups of KTRs when compared to those of controls, showing lower peak VO_2_, maximal power output, VO_2_ at the VT1 and peak heart rate. The VO_2_/Work Rate slope was, however, comparable between all groups (see Table 3).

**Table 3:** Cardiopulmonary exercise test parameters and VO_2_ kinetics analysis

	3-month group	12-month group	Control group
Number	21 (13 M, 8 F)	14 (12 M, 2 F)	16 (9 M, 7 F)
Peak parameters			
Peak VO_2_ (mL/min)	1443.81 ± 374.90*^,^**	1951.58 ± 591.74**	2861.49 ± 771.90
Peak VO_2_ (mL/kg/min)^a^	21.30 ± 4.34**	26.37 ± 7.96**	41.70 ± 7.82
Percentage of the predicted VO_2_ peak (%)	75.7 ± 15.5**	83.7 ± 22.8**	109.9 ± 18.7
Maximal power output (W)^a^	105.48 ± 28.19**	148.21 ± 48.22**	251.56 ± 58.79
Peak heart rate	133.43 ± 20.56**	144.07 ± 21.41**	181.25 ± 10.40
Percentage of the maximal predicted heart rate (%)	79.67 ± 12.16**	84.29 ± 11.49**	93.38 ± 5.21
Peak Respiratory exchange ratio^a^	1.18 ± 0.10**	1.18 ± 0.04**	1.27 ± 0.09
Parameters at the first ventilatory threshold			
VO_2_ (mL/min)^a^	910.43 ± 179.27**	1090.86 ± 345.92**	1594.63 ± 514.27
VO_2_ (mL/kg/min)^a^	13.50 ± 2.30**	14.74 ± 4.50**	23.44 ± 6.42
VO_2_ (percentage of peak VO_2_)	64.14 ± 7.63	56.88 ± 11.21	56.33 ± 11.54
Power output (W)^a^	50.95 ± 15.70**	70.00 ± 33.29**	123.44 ± 44.22
VO_2_ kinetics analysis			
Time constant “tau” (τ) (s)	50.40 ± 13.11**	43.84 ± 11.57**	28.91 ± 8.37
Other parameters			
VE/VCO2 slope^a^	29.28 ± 4.25**	26.89 ± 2.57	24.91 ± 2.93
Oxygen uptake efficiency slope (mL/logL)	1561.14 ± 375.02**	1904.05 ± 477.45**	2762.49 ± 654.78
VO_2_/Work slope (mL/W)	8.77 ± 2.0	10.04 ± 1.73	9.48 ± 1.42

Parameters are expressed as mean ± SD

*Significantly different (*P* < .05) from value obtained in 12-month group, **Significantly different (*P* < .05) from value obtained in control group

^a^Data are non-normally distributed in at least one of the three groups

When comparing both groups of KTRs, aerobic exercise capacity was higher 12 months after transplant compared to 3 months post-surgery. In particular, peak VO_2_ (21.30 ± 4.34 vs 26.37 ± 7.96 ml/min/kg; *P* = 0.043), maximal power output (105.48 ± 28.19 vs 148.21 ± 48.22 W; *P* = 0.007) and OUES (1561.14 ± 375.02 vs 1904.05 ± 477.45; *P* = 0.023) were found higher 1 year post-surgery. This was also confirmed by a more efficient ventilation as shown by the lower VE/VCO_2_ slope 12 months after transplantation (29.28 ± 4.25 vs 26.89 ± 2.57, *P* = 0.048; see Table 3 and Table S1 of the supplementary material).

**Fig. 2:**
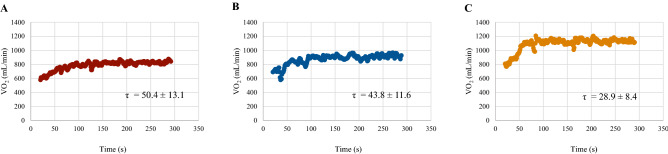
The average values of VO_2_, plotted against time and recorded during the constant load phases in the three study groups. Panel A shows the VO_2_ kinetics of kidney transplant recipients evaluated 3 months after surgery, panel B shows VO_2_ kinetics of patients evaluated 1 year after surgery and panel C shows VO_2_ kinetics of the control group. The respective average time constant (τ) values of VO_2_ kinetics are reported

Finally, the time constant τ of the VO_2_ kinetics was slower in KTRs compared to controls (50.40 ± 13.11 s, 95% CI 44.4–56.4 s; 43.84 ± 11.57 s, 95% CI 37.2–50.5 s; 28.91 ± 8.37 s, 95% CI 24.4–33.4 s; both *P* < 0.01) (Table 3, Fig. 2).

**Fig. 3:**
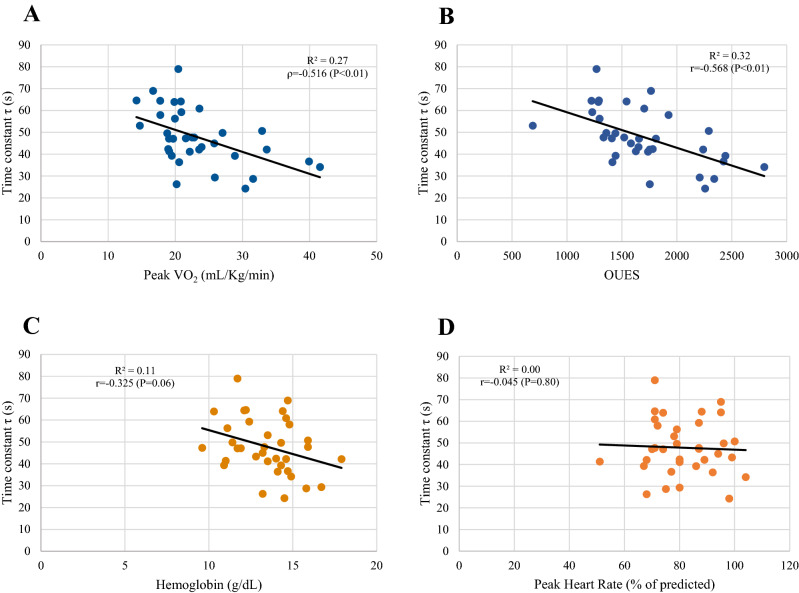
The time constant τ of KTRs is negatively correlated with parameters of aerobic fitness, such as VO_2_/kg (panel A) and the oxygen uptake efficiency slope (panel B). However, the time constant τ seems less associated with patients’ blood Hb concentration and peak heart rate (panel C and D, respectively)

Moreover, the time constant τ of KTRs showed negative correlations with peak VO_2_ (ρ = − 0.52, *P* < 0.01), peak power output (ρ = − 0.59; *P* < 0.01), VO_2_ at the VT1 (ρ = − 0.41; *P* = 0.01), power output at the VT1 (ρ = − 0.71; *P* < 0.01) and the OUES (*r* = − 0.57; *P* < 0.01). No significant correlation was found between τ and Hb (*r* = − 0.33, *P* = 0.06) or percentage-predicted peak heart rate (HR) (*r* = − 0.05, *P* = 0.80). Conversely, Hb was more strongly correlated with main parameters of cardiorespiratory fitness, similarly to what found for patients’ peak HR (see Fig. 3 and Table S2).

## Discussion

To the authors’ knowledge, this is the first study to evaluate specifically VO_2_ kinetics in KTRs with different follow-up periods after transplantation. The aim underlying this study was thus to investigate non-invasively the peripheral limitations to physical exercise in a group of patients with alterations at the muscular and microvascular level.

KTRs are known to have reduced aerobic exercise capacity in terms of peak VO_2_, which tends to show a partial recovery during time after transplant (Kempeneers et al. 1990; Habedank et al. 2009; Painter et al. 2011). The present study supports this evidence, in fact patients evaluated early after transplant showed an abnormally low peak VO_2_ (75.7 ± 15.5% of predicted). This value seems to improve 1 year after transplant (83.7 ± 22.8% of predicted; Table S1). These results confirm existing data on VO_2_ improvement after kidney transplant (Habedank et al. 2009; Painter et al. 2011) and, given the cross-sectional design of this study, warrant further longitudinal evaluations on larger numbers of patients. It seems reasonable that the main contributors to exercise intolerance in these patients have to be individually evaluated, considering central and peripheral limitations of the oxygen transport system. CKD patients often carry several complications of end-stage renal disease, such as anaemia, autonomic dysfunction, peripheral vascular disease and muscular abnormalities (Painter et al. 2011). The improvement in exercise capacity seen after kidney transplant has been associated with an increased cardiac output secondary to an increased peak heart rate (Painter et al. 2011), while the contribution of haemoglobin is likely to play a minor role (Marrades et al. 1996; Painter et al. 2011). Peripheral limitations typical of these patients seem to contribute to the impairment of aerobic capacity. Indeed, Painter et al. found no improvement in peripheral oxygen extraction of KTRs during a maximal exercise test compared to the pre-transplant evaluation, suggesting that no changes occur in muscle oxidative capacity with the normalization of renal function (Painter et al. 2011). Similar results have been found also in patients with CKD, where the improvement in haematocrit and cardiac output obtained with erythroid-stimulating agents and exercise training was not sufficient to normalize the patients’ oxygen consumption, likely due to abnormalities found at the muscular level (Stray-Gundersen et al. 2016). Thus, the evaluation of muscular oxidative capacity in patients with CKD or KTRs appears of primary importance for the assessment of physical function.

Our results showed that the two groups of KTRs had slower VO_2_ kinetics compared to a control group of young and healthy adults, and that these higher time constants were correlated with worse cardiorespiratory fitness, suggesting a contribution of peripheral limitations to these patients’ exercise capacity. Even though few data on KTRs are available for comparisons, the time constants of our population and the control group appear to be in line with previous evidence on VO_2_ kinetics, indicating feasibility and reproducibility in clinical settings (Tomczak et al. 2008). Moreover, George et al. analysed the VO_2_ time constants in a group of healthy individuals aged 18–45 year old that performed physical activity 2–4 times per week and values determined in the control group of the present study are similar to the ones they found (τ = 28.91 ± 8.37 s vs 26.8 ± 7.5 s, respectively) (George et al. 2018). Compared to the control group, the time constants of KTRs were significantly slower at 3 and 12 months after transplantation (50.4 ± 13.11 s and 43.84 ± 11.57 s, respectively). Although methodological differences between studies must be considered, VO_2_ kinetics of KTRs 3 months after surgery seemed generally slower than those of older inactive individuals (44.8 ± 10.9 s), which were comparable for KTRs of the 12-month group (George et al. 2018). However, despite the age heterogeneity between groups, it was shown that physical fitness and not aging per se, seems to determine the response of VO_2_ kinetics (George et al. 2018). Moreover, even though data about VO_2_ kinetics in KTRs are limited, the values of time constants previously found in patients with CKD undergoing dialysis (62.5 ± 19.6 s) (Reboredo et al. 2015), let us to hypothesize a partial recovery of submaximal peripheral aerobic metabolism after transplant, associated with the shown improved cardiorespiratory fitness after kidney transplantation. However, further longitudinal studies are needed to ultimately assess the changes in VO_2_ kinetics from end-stage renal disease to a long-term follow-up after renal transplantation.

Moreover, among patients of the present study, VO_2_ kinetics were strongly correlated with indicators of physical fitness, and showed no significant correlations with central determinants of the cardiopulmonary response to exercise such as Hb and peak HR. On the contrary, indicators of physical fitness showed better correlations with Hb and peak HR (Table S2). Although these data cannot provide information regarding the underpinning pathophysiological mechanisms affecting VO_2_ kinetics, study outcomes may suggest that also peripheral adaptations occur after kidney transplantation. As previously mentioned, current evidence supports the hypothesis that slowed VO_2_ kinetics during moderate intensity cycling mainly reflect an impaired oxidative capacity of the muscle. Even if this assumption is still debated, particularly for patients with chronic diseases (Poole and Jones 2012), the results of the present study are in agreement with previous study outcomes, suggesting that VO_2_ kinetics could provide additional information about these patients’ peripheral response to exercise (Tomczak et al. 2008). Furthermore, the found correlations and reproducible absolute τ values when compared with previous studies show that an evaluation of VO_2_ kinetics inside a clinical setting, with a pre-defined constant load, can provide reliable values of time constants. Moreover, these outcomes seem to accurately reflect experimental evaluations of VO_2_ kinetics at a defined percentage of the previously assessed VT1. As previously stated also by Reboredo et al. in relation to patients with CKD, the assessment of VO_2_ kinetics provides additional information about an exercise intensity domain that is close to that of most physical activities of daily living (ADLs) (Reboredo et al. 2015). On these bases, the analysis of VO_2_ kinetics could thus result as a useful integration to the comprehensive functional evaluation of KTRs’ physical fitness, being relatively effort-independent. Indeed, the time constant during submaximal exercise could be used as marker for peripheral dysfunction in patients whose exercise tolerance is limited by muscular abnormalities (Stray-Gundersen et al. 2016).

Finally, although the absolute values of the time constants of KTRs got closer to those of the control group during 12 months of follow-up, a statistically significant difference was not reached between both groups of patients. Taking also into account that peak VO_2_ was higher 12 months after transplantation, this might suggest a slower recovery of peripheral muscle metabolism revealed at submaximal exercise. Considering the good responsiveness of VO_2_ kinetics to exercise training programs, such type of intervention should be recommended to KTRs to improve their peripheral exercise tolerance, especially at the clinically significant workloads of common ADLs (Reboredo et al. 2015).

### Limitations and perspectives

The limitations of the current study are mainly due to the cross-sectional design, which does not allow exhaustive considerations on the time course of VO_2_ kinetics in KTRs. Furthermore, this study has not been designed to specifically investigate underlying pathophysiological mechanisms but study outcomes should motivate future basic research to address this issue. Moreover, future studies should investigate how ageing and exercise test intensity may affect VO_2_ kinetics in this population. A prospective randomized controlled study, with a healthy and sedentary age-matched control group, investigating the impact of a specific exercise training intervention on VO_2_ kinetics, would provide interesting information on the clinical impact and reversibility of peripheral exercise limitations.

### Conclusions

In conclusion, it can be stated that our study is the first to evaluate peripheral oxidative muscle metabolism by VO_2_ kinetics with specific standardized exercise testing in KTRs. It has been shown that KTRs have impaired exercise tolerance and physical fitness, with a partial although incomplete recovery 1 year after transplant. Also, the time constant τ of VO_2_ kinetics is slower in KTRs at 3 and 12 months after transplantation compared to young and healthy subjects. The reduced aerobic exercise capacity of KTRs strongly correlated with slower VO_2_ kinetics, which seem less associated with central determinants. A clinical evaluation of VO_2_ kinetics could add useful information to routine cardiopulmonary exercise testing of KTRs, likely reflecting peripheral pathophysiological aspects of the integrated response to physical exercise, also investigating the workload intensities crucial for activities of daily living in this population.

## Supplementary Information

Below is the link to the electronic supplementary material.

Supplementary file1 (DOCX 20 KB)

## Data Availability

The datasets generated during and/or analysed during the current study are available from the corresponding author on reasonable request.

## Abbreviations

ANOVA: Analysis of variance
CKD: Chronic kidney disease
Hb: Haemoglobin
HR: Heart rate
KTRs: Kidney transplant recipients
OUES: Oxygen uptake efficiency slope
VO_2_ kinetics: Kinetics of oxygen uptake
VT1: First ventilatory threshold
