# Supplementary material for: A clinical evaluation of VO2 kinetics in kidney transplant recipients
Source: Eur J Appl Physiol. 2021 Apr 3;121(7):2005–13. doi: 10.1007/s00421-021-04672-x (PMC8192378; doi:10.1007/s00421-021-04672-x)
Supplement: Supplementary file 1 — Supplementary file1 (DOCX 20 KB) [file 421_2021_4672_MOESM1_ESM.docx]

SUPPLEMENTARY MATERIAL

| **Table S1**. Cardiopulmonary exercise test parameters and VO_2_ kinetics analysis in the two groups of KTRs. | | | |
| --- | --- | --- | --- |
|  | 3-month group | 12-month group | P |
| Number | 21 (13 M, 8 F) | 14 (12 M, 2 F) |  |
| *Peak parameters* |  |  |  |
| Peak VO_2_ (mL/min) | 1443.81 ± 374.90 | 1951.58 ± 591.74 | .004 |
| Peak VO_2_ (mL/kg/min) | 21.30 ± 4.34 | 26.37 ± 7.96 | .043 |
| Percentage of the Predicted VO_2_Peak (%) | 75.73 ± 15.53 | 83.72 ± 22.79 | .225 |
| Maximal power output (Watt) | 105.48 ± 28.19 | 148.21 ± 48.22 | .007 |
| Peak Heart Rate | 133.43 ± 20.56 | 144.07 ± 21.41 | .149 |
| Percentage of the Maximal Predicted Heart Rate (%) | 79.67 ± 12.16 | 84.29 ± 11.49 | .269 |
| Peak Respiratory Exchange Ratio^ | 1.18 ± 0.10 | 1.18 ± 0.04 | .752 |
| *AT parameters* |  |  |  |
| VO_2_ at the Anaerobic Threshold (mL/min) | 910.43 ± 179.27 | 1090.86 ± 345.92 | .051 |
| VO_2_ at the Anaerobic Threshold (mL/kg/min) | 13.50 ± 2.30 | 14.74 ± 4.50 | .290 |
| VO_2_ at the Anaerobic Threshold (percentage of peak VO_2_) | 64.14 ± 7.63 | 56.88 ± 11.21 | .046 |
| Power output at the Anaerobic Threshold (Watt)^ | 53.57 ± 13.34 | 72.14 ± 32.15 | .066 |
| *VO_2_ kinetics analysis* |  |  |  |
| Time constant “tau” (τ) (seconds) | 50.40 ± 13.11 | 43.84 ± 11.57 | .139 |
| *Other parameters* |  |  |  |
| VE/VCO2 Slope^ | 29.28 ± 4.25 | 26.89 ± 2.57 | .048 |
| Oxygen Uptake Efficiency Slope (mL/logL) | 1561.14 ± 375.02 | 1904.05 ± 477.45 | .023 |
| VO_2_/Work Slope (mL/Watts) | 8.77 ± 2.00 | 10.04 ± 1.73 | .060 |
| Parameters are expressed as mean ± SD. ^ non-normally distributed in at least one of the two groups. | | | |

| **Table S2**. Study correlations | | | | | | | | | |
| --- | --- | --- | --- | --- | --- | --- | --- | --- | --- |
|  | Hb | Peak VO_2_ (mL/Kg/min)^ | Peak power output^ | Peak HR  (% pred.) | VO_2_ AT (mL/min/Kg)^ | Power output at the AT ^ | VO_2_/Work Slope (mL/Watt) | OUES | Tau |
| Hb |  |  |  |  |  |  |  |  |  |
| Peak VO_2_ (mL/min/Kg)^ | .439** |  |  |  |  |  |  |  |  |
| Peak power output^ | .645** | .718** |  |  |  |  |  |  |  |
| Peak HR (% pred.) | - | .426* | - |  |  |  |  |  |  |
| VO_2_ AT (mL/min/Kg)^ | - | .740** | .481** | .499** |  |  |  |  |  |
| Power output at the AT ^ | .466** | .585** | .819** | - | .568** |  |  |  |  |
| VO_2_/Work Slope (mL/Watt) | .431** | 499** | .578** | - | - | .356* |  |  |  |
| OUES | .552** | .550** | .836** | - | .415** | .759** | .655** |  |  |
| Tau | - | -.516** | -.590** | - | -.411* | -.713** | - | -568** | - |
| Correlations are expressed as Pearson’s (r) or Spearman’s (ρ) correlation coefficient, as appropriate. *correlation is significant at the 0.05 level, ** correlation is significant at the 0.01 level. Hb, hemoglobin; HR, heart rate; AT, anaerobic threshold; OUES, oxygen uptake efficiency slope. ^ non-normally distributed data. | | | | | | | | | |
